# Supplementary material for: Chromothripsis during telomere crisis is independent of NHEJ, and consistent with a replicative origin
Source: Genome Res. 2019 May;29(5):737–49. doi: 10.1101/gr.240705.118 (PMC6499312; doi:10.1101/gr.240705.118)
Supplement: Supplemental Material [file supp_gr.240705.118_Supplemental_file_1.zip › contigs/annotated_contigs/DB75/contig.2.DB75_length_519_mean_cov_6.71098265896.docx]

**DB75_length_519_mean_cov_6.71098265896**

```
GTAAATTAGTTCAACCATTGTGGAAGACAGAGTGGCAATTCCTCAAGGATTTAGAACTAGAAATACCATTTGACCCAGCAATCCCATTA
 >chr10:85605083-85605430 - E=2e-197
CTGGGTATATATCCAAAGGATTATAATTCATTCTACTATAAAGACACATGCACACGTATGTTTATTGCAGCACTATTCACAATAGCAAA

GACTTGGAACCAACCCAAATGCCCATCAATGATAGGCTGGACAAAGAAAATATGGCACATATACACCATGGAATACTATGCAGCCATAA

AAAAGGATCAGTTCATGTCCTTTGCAGGGACATAGATGAAGCTGGAAACCATCATTCTAAGCAAATTATCACAAGGACAG|G|ATATAT
                                                                                  >chr1
ATATATCTTAGTTTCATTGAATTTTTTCCATTTATTGGATTTGAATTTCATTTCATTGAATTTTTCCATTTATATATTCTTTATTTCAT
0:85604149-85604320 - E=3e-88
TTATTTCTGTGCTAATCTTTCTTACTTCCCTCCTTCTGATAACTTTGGGTTTAGTTTGTTCCTCATTTTCTAGTTC
```
